# Chronic restraint stress promotes oral squamous cell carcinoma development by inhibiting ALDH3A1 via stress response hormone

**DOI:** 10.1186/s12903-023-03787-1

**Published:** 2024-01-08

**Authors:** Shihong Luo, Huiqing Long, Fangzhi Lou, Yiyun Liu, Haiyang Wang, Juncai Pu, Ping Ji, Xin Jin

**Affiliations:** 1 College of Stomatology, Chongqing Medical University, Chongqing, 401147 China; 2 Chongqing Key Laboratory of Oral Diseases and Biomedical Sciences, Chongqing, 401147 China; 3 NHC Key Laboratory of Diagnosis and Treatment On Brain Functional Diseases, The First Affiliated Hospital of Chongqing Medical University, Chongqing, 400042 China

**Keywords:** Chronic restraint stress, ALDH3A1, Hormone, Oral squamous cell carcinoma, Mitochondrial metabolism

## Abstract

**Background:**

Chronic restraint stress (CRS) has iteratively been reported to be possibly implicated in the development of numerous cancer types. However, its role in oral squamous cell carcinoma (OSCC) has not been well elucidated. Here we intended to evaluate the role and mechanism.

**Methods:**

The effects of CRS were investigated in xenograft models of OSCC by using transcriptome sequencing, LC–MS, ELISA and RT-PCR. Moreover, the role of CRS and ALDH3A1 on OSCC cells was researched by using Trans-well, flow cytometry, western blotting, immunofluorescence, ATP activity and OCR assay. Furthermore, immunohistochemical staining was employed to observe the cell proliferation and invasion of OSCC in xenotransplantation models.

**Results:**

CRS promoted the progression of OSCC in xenograft models, stimulated the secretion of norepinephrine and the expression of ADRB2, but decreased the expression of ALDH3A1. Moreover, CRS changed energy metabolism and increased mitochondrial metabolism markers. However, ALDH3A1 overexpression suppressed proliferation, EMT and mitochondrial metabolism of OSCC cells.

**Conclusion:**

Inhibition of ALDH3A1 expression plays a pivotal role in CRS promoting tumorigenic potential of OSCC cells, and the regulatory of ALDH3A1 on mitochondrial metabolism may be involved in this process.

**Supplementary Information:**

The online version contains supplementary material available at 10.1186/s12903-023-03787-1.

## Background

Oral squamous cell carcinoma is the most frequent malignant tumor of the head and neck with potential for lymph node metastasis, deep invasion and high recurrence rate, which shows poor prognosis and high mortality [1, 2]. Despite the constant progression of therapeutic approaches, the 5-year survival rate has stagnated at about 50% [3]. Hence, illustration of the molecular mechanisms related to OSCC is primarily important to development of original theragnostic plans.

Chronic stress is an ineluctable part of life, and people have always experienced undue stress caused by global issues [4]. Occupational stress and abnormal adversities are the main sources of chronic stress. Chronic stress underlying negative effects include anxiety, insomnia and depression [5], and also increasing the risk of mental disease and carcinomatosis [6]. Chronic stress can alter immune capabilities and regulate the advancement of certain neoplasms by stimulating the hypothalamic–pituitary–adrenal (HPA) axis and freeing neurotransmitter which acts on adrenergic receptors [7, 8]. Study has revealed that patients with OSCC have an upper incidence of depression and anxiety than normal, and that psychiatric disorders are strongly associated with survival and treatment outcomes of patients with OSCC [9].

Neurohormonal products originated in chronic stress can affect the behavior of oral cancer cells [10]. Studies have reported that neurohormone participates in the processes of invasion and advancement of different types of cancer via beta-adrenergic signaling [11–13]. In addition, the increased of norepinephrine (NE) level in microenvironment is predictive for OSCC occurrence [14]. Whereas, whether chronic stress influences the development of oral squamous cell carcinoma through stress response hormone, the potential mechanism about it remains unclear.

Aldehyde dehydrogenase 3A1 (ALDH3A1), an important member of the ALDHs superfamily, regulates cellular function by affecting the metabolism [15]. ALDH3A1 is highly expressed in oral epithelium, nasal epithelium and small salivary gland [16, 17]. Recent research has showed that the expression of ALDH3A1 was upregulated in several cancer types and demonstrated clear association between ALDH3A1 and cancer progression [18]. However, another study reported that the expression of ALDH3A1 was decreased in OSCC tissue and the low expression of ALDH3A1 is connected with inferior prognosis of patients [19]. Although ALDH3A1 is known to regulate cell function and cancer prognosis, the effects and underlying mechanisms of chronic stress states remain unclear. In this study, we examined the influence of CRS on OSCC progression, and systematically investigated the role of ALDH3A1 in OSCC cells proliferation and invasion.

## Methods

### Cell culture

The human OSCC cell lines (HN6 and HSC4) were provided by ATCC (Manassas, USA). All cells were cultured in DMEM of Gibco (Carlsbad, USA) with 10% FBS provided by Invitrogen (Carlsbad, USA).

### Tumor xenografts and CRS model

All experiments of animal were approved and supervised by the Ethics Committee of the College of Stomatology, Chongqing Medical University (Approval No. 2021063), and all methods were performed in accordance with the relevant guidelines and regulations. Four-week-old athymic nude mice were acquired by Cavensbiogle (Suzhou, China). All mice were housed under SPF condition. All mice were randomly departed into control (Con) and CRS group, and each group contained six mice. For CRS group, mice were blocked in 50 ml centrifuge tube for 2 h a day for 28 days. After one day of CRS, HN6 cells (2 × 10^6^) were injected subcutaneously to the left flanks skin [20]. The tumor size was measured once a week with vernier calipers, and tumor volumes were computed according to the coming formula: V = 1/2 × a × b^2^ (V = volume, a = long diameter, and b = short diameter). After 5 weeks of initial CRS, all mice in the CRS and Con groups were euthanized with carbon dioxide. We collected serum and tumors for further analysis, and recorded the weights of excised tumor tissues. All swatches were analyzed by transcriptomics, metabolomics Enzyme linked immunosorbent assay (ELISA).

### Behavioral assessment

Behavioral tests on mice included OFT, TST and FST. Then, video tracking equipment of SMART (Barcelona, Spain) was used for quantitative analysis [21]. The tracking software system recorded the motion trajectory and counted the travel distance and rest time, and the EthoVision XT 13.0 software was used for analysis. The specified procedures are described in our previous study [20].

### Measurement of NE

The plasma was collected for testing [20]. Detection of NE level by ELISA kit (JL13969-96 T, Shanghai, China). The measurement was conducted in a single-blinded manner.

### Transcriptomics

Total RNA was abstracted from tumor samples with Trizol reagent of Invitrogen (Carlsbad, USA). And RNA integrity was determined by using Agilent 2100 Bioanalyzer of Agilent (CA, USA). Then, RNA samples were sent to platform of Major (Shanghai, China) for NGS analysis. The datasets are available in the cloud.majorbio.com, https://cloud.majorbio.com/page/v2/project/task.

### Metabolomics

Tumor tissues were prepared for metabolomics analysis by liquid chromatography–mass spectrometry (LC–MS). The metabolites were extracted and analyzed by Major (Shanghai, China). The specified procedures were afforded in the Supplementary. The datasets generated during the current study are available in the cloud.majorbio.com, https://cloud.majorbio.com/page/v2/project/task.

### Bioinformatics seeking

The premier metabonomic datum were imported into the XCMS program for analysis, and data missing more than 1/2 of metabolites were excluded. The strategy of PCA analysis in this study was to screen the differential metabolites between groups based on Student’s t-test. Metabolites with a value of *p* < 0.05 and VIP (variable importance value, VIP) > 2 among the top 20 expression levels were selected as significant metabolites. R software was used in combination with KEGG and MetaboAnalyst 4.0 (http://www.MetaboAnalyst.ca/) for metabolic pathway analysis.

### RT-PCR

Total RNA was extracted from tumor tissues and treated cells by using RNA extraction kit of Beyotime (Shanghai, China). cDNA was synthesized from total RNA by using Prime Script RT reagent kit of MCE (Shanghai, China). The Real-time PCR was performed by using ABI 7300 system of Biosystems (CA, USA) via SYBR Premium ExTaq kit of MCE (Shanghai, China). Quantity of gene was planned using method 2^−ΔΔCt^ and normalized to β-actin. Detailed procedures and primer sequences for RT-PCR were listed in supplementary methods and supplementary Table 1.

### Western blot

The total protein was extracted from the treated OSCC cells by using RIPA buffer with protease inhibitor (Roche, Basel, Switzerland). The blots were cut prior to hybridization with antibodies during blotting. The detailed protocols, primary antibodies, and full-length original blots are provided in the supplementary.

### Cell proliferation

The ability of cell proliferation was explored by using Cell Counting Kit-8 (Tokyo, Japan). Specific procedures were provided in supplementary.

### Flow cytometry

The apoptosis and cycle of treated OSCC cells were treated by flow cytometry (BD FACSCanto). Detailed programs were listed in the supplementary.

### Cell invasion and migration

The invasive ability of cells was surveyed by transwell assay, and the migration talent of cells was examined by scratch wound healing test. Specified procedures were provided in the supplementary.

### Immunofluorescence

Immunofluorescence (IF) was performed on treated cells, as previously reported [20]. Detailed programs were listed in the supplementary. methods and supplementary Table 1.

### Lentiviral transfection

Human ALDH3A1 lentiviral and negative control lentivirus were produced by QingKe (Shanghai, China). ALDH3A1 overexpressed lentiviral was transfected into HN6 and HSC4 cells pursuant to the manufacturer's protocol to construct stable cell lines.

### Immunohistochemistry

The transplanted tumor tissues were analyzed by immunohistochemistry (IHC). Detailed programs were supplied in the supplementary.

### Adenosine triphosphate (ATP) activity assay

Lv-ALDH3A1 and Lv-Con HN6 cells ATP contents were detected using ATP bioluminescence assay kit of Beyotime (Shanghai, China), pursuant to the manufacturer’s instruction.

### Oxygen consumption rate (OCR) Assay

Oxygen consumption rate of Lv-ALDH3A1 and Lv-Con HN6 cells were measured with the Seahorse XFe96 Analyzer (Agilent Technologies, Inc) and Extracellular Oxygen Consumption Assay Kit of Abcam (no. ab197243). The cell inoculation density was 5 × 10^4^ per well. Metabolic inhibitors in the assay included oligomycin, rotenone, and antimycin A. After measurement, data was analyzed with Wave software (Agilent Technologies, Inc).

### Statistical analysis

Data were expressed as the mean ± SD. All statistical analyses were performed with GraphPad 8.0 and SPSS25.0. The statistical significance of differences between two groups was analyzed using two-tailed Student’s t-tests. The differences were considered statistically significant at *p* < 0.05.

## Results

### Chronic restraint stress induces psychiatric symptoms, stimulates hormone secretion, and promotes tumor growth of OSCC in xenograft models

**Fig. 1:**
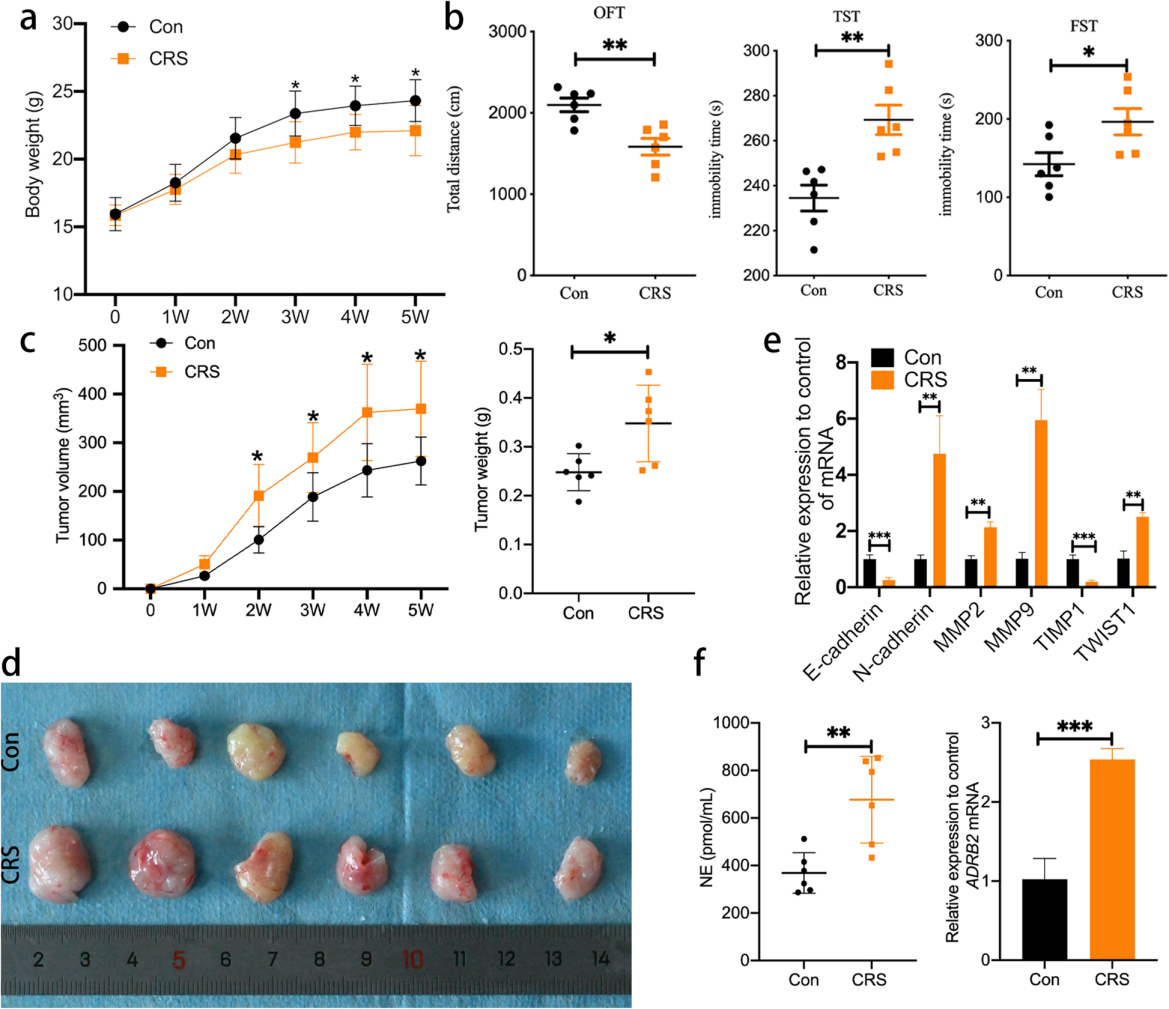
Chronic restraint stress induced psychiatric symptoms, stimulate hormone secretion, and promote tumor growth. **a** Body weight curve of Con and CRS groups. **b** Results of behavioral assessment, include open field test (OFT), tail suspension test (TST) and forced swimming test (FST). **c** Growth curves of tumor volume and weight of tumor nodules. **d** Tumor nodules of both groups. **e** mRNA expression of tumor regulatory factors and EMT-related markers in mice xenografts. **f** Levels of plasma NE and mRNA expression of ADRB2 in tumor tissue. Data are expressed as mean ± SD (*n* = 6); *, *P* < 0.05; **,* P* < 0.01; ***, *P* < 0.001

To explore the effects of CRS on OSCC in vivo, HN6 cells were transplanted subcutaneously into nude mice. The treatment group mice began CRS one day before the injection of HN6. Four weeks later, the weight of the mice under CRS was significantly lessened (Fig. 1a). Compared with Con group, the center motion distance of OFT was significantly decreased and the rest time of FST and TST in CRS group was expressively increased (Fig. 1b). The tumor volumes and masses of the mice in CRS group were significantly bigger than those of Con group (Fig. 1c, d). Then, to determine whether CRS promoted the invasion of OSCC, we detected the expression of EMT related markers. RT-PCR results revealed that the expressions of N-cadherin, MMP2, MMP9 and Twist1 were up-regulated in the CRS group, while the expressions of E-cadherin and TIMP1 were down-regulated (Fig. 1e). Notably, the concentration of norepinephrine (NE) in plasma of mice in CRS group was expressively higher than that of Con group and the expression of ADRB2 was upregulated in CRS group (Fig. 1f). The above data indicated that CRS induces psychiatric symptoms, increases NE secretion and enhances OSCC growth and invasion.

### Chronic restraint stress promotes the carcinogenic potential of OSCC cells in vitro

**Fig. 2:**
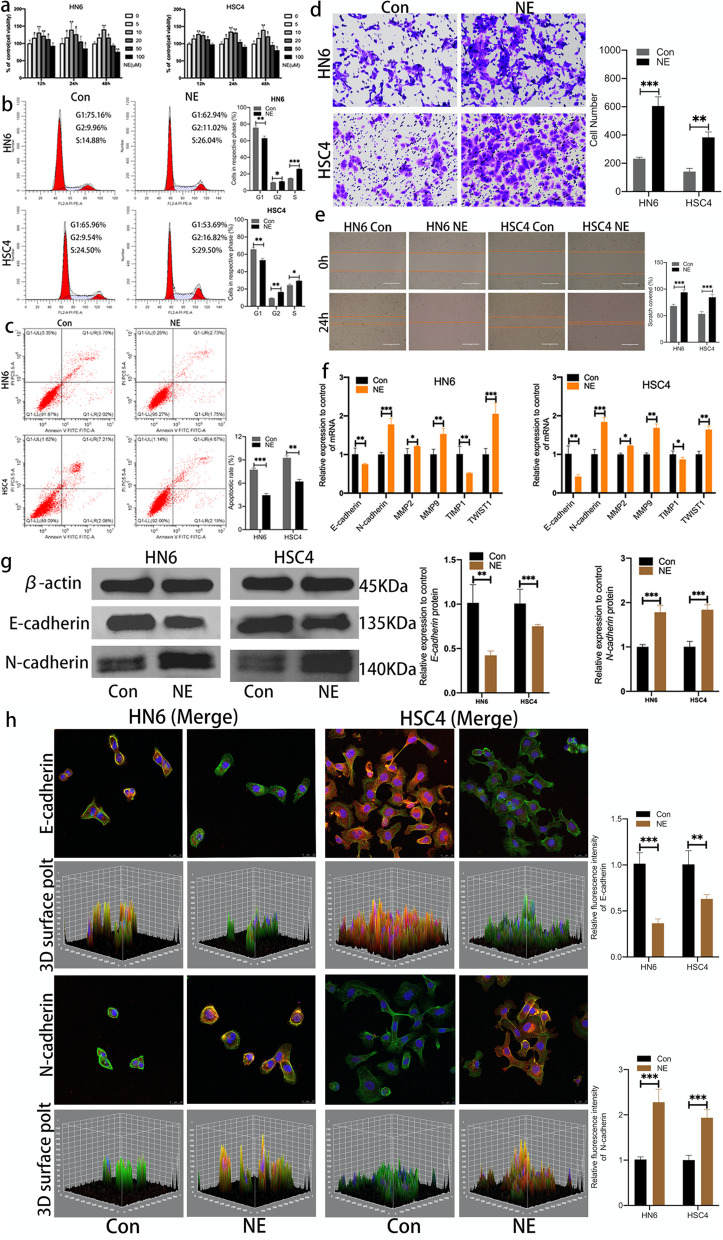
Chronic stress promotes the carcinogenic potential of OSCC cells in vitro. **a** Effects of NE of different concentrations on the proliferation potential of HN6 and HSC4 cell lines were determined by CCK8 assay. **b** Rate of cell cycle was determined by flow cytometry. **c** Rate of apoptosis in both groups. **d** Cell invasion ability was determined using trans-well assays. Scale bars, 200 μm. **e** Cell migration ability was determined using wound-healing assays. Scale bars, 400 μm. **f** mRNA levels of tumor regulatory factor and EMT-related markers were determined by RT-qPCR of Con and NE groups. **g** Protein levels of EMT-related markers were determined by western blot (The blots were cropped and the full-length raw blots are provided in the supplementary). **h** Confocal laser scanning microscope images of immunofluorescent staining of E-cadherin and N-cadherin (EMT-related markers), blue for nuclear, green for cytoskeleton and red for protein. Scale bars, 25 μm. Data are expressed as mean ± SD (*n* = 3); *, *P* < 0.05; **, *P* < 0.01; ***, *P* < 0.001

Based on the fact that norepinephrine (NE) is a key physiological argument of chronic stress [22], we asked whether that NE secreted in the CRS state directly promotes OSCC cells proliferation. HN6 and HSC4 cells were served with individual concentrations of NE for 12, 24 and 48 h, respectively. As shown in (Fig. 2a), CCK-8 results revealed that NE significantly promoted the propagation of HN6 and HSC4 cells. After treated with 10um NE for 24 h, the cell proliferation reached the peak, so we used the 10um of NE for the next research. Furthermore, flow cytometry analysis revealed that the percentage of cells in S-phase were significantly increased and the percentage of apoptotic cells were evidently decreased in NE group (Fig. 2b, c). Moreover, the effect of norepinephrine on the invasive phenotypes for HN6 and HSC4 cells were valued by transwell invasion assay and wound healing experiment, after therapy with NE the OSCC cells also exhibited remarkably higher capability of invasion and migration (Fig. 2d, e). In addition, we furtherly spotted the expression of EMT-related marker through a series of experiments. RT-PCR showed upregulation of N-cadherin, MMP2, MMP9 and TWIST1 while downregulation of E-cadherin and TIMP1 in NE treatment group (Fig. 2f). Western blot and immunofluorescence staining showed that N-cadherin was up-regulated while E-cadherin down-regulated after NE treatment (Fig. 2g, h, sFig 1). Taken together, CRS can enhance the growth, transplantation and invasion of OSCC cells via stress response hormone.

### Chronic restraint stress suppresses the expression of ALDH3A1

**Fig. 3:**
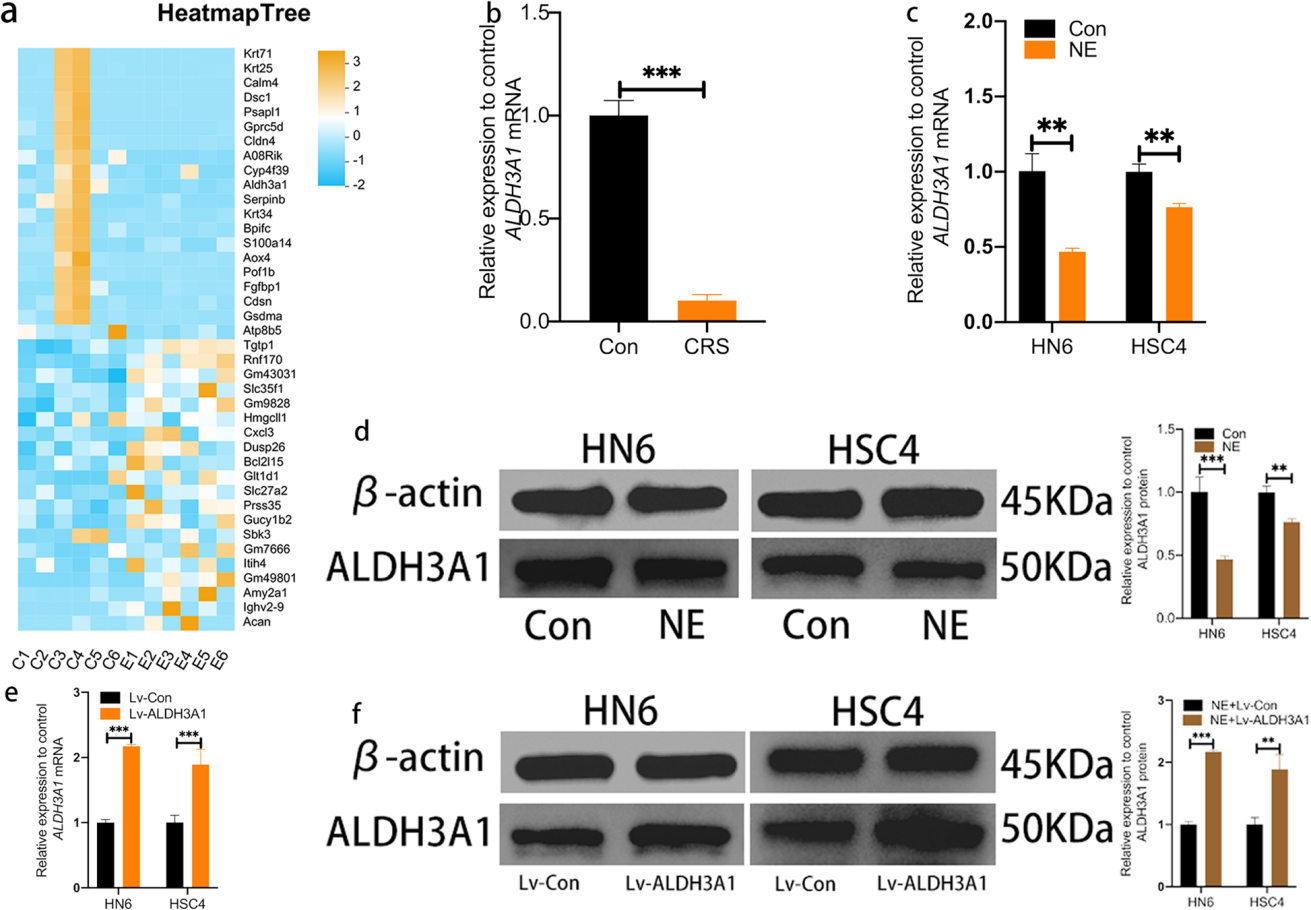
CRS suppressed the expression of ALDH3A1. **a** Heatmap Tree of RNA-seq (*n* = 6). **b** mRNA expression of ALDH3A1 in mice xenograft tumors. **c** mRNA expression of ALDH3A1 in HN6 and HSC4 of Con and NE groups. **d** Protein levels of ALDH3A1 in Con and NE groups of OSCC cells (The blots were cropped and the full-length raw blots are provided in the supplementary). **e** mRNA expression of ALDH3A1 in OSCC cells with Lv-con or Lv-ALDH3A1 treatment. **f** Protein levels of ALDH3A1 in Lv-con and Lv-ALDH3A1 groups of OSCC cells (The blots were cropped and the full-length raw blots are provided in the supplementary). Data are expressed as mean ± SD (*n* = 3); *, *P* < 0.05; **, *P* < 0.01; ***, *P* < 0.001

To trace the underlying molecular mechanisms by which CRS promoted carcinogenic potential of OSCC cells, we performed transcriptome sequencing on tumor samples. The top 20 genes with up-regulated and down-regulated differential expression were screened (Fig. 3a). Antecedent researches have reported that ALDH3A1 plays a key role in the malignant behavior of cancers through stimulating signaling pathways [15, 18, 19, 23]. Our results also identified that ALDH3A1 was significantly downregulated in CRS group (Fig. 3b). Moreover, ALDH3A1 mRNA and protein levels were decreased by stress response hormone in vitro (Fig. 3c, d). These data have implicated that ALDH3A1 is a key factor modulated by CRS. To clarify the function of ALDH3A1, we established transfected cells overexpressing with lentiviral. And transfected effect was validated by RT-PCR and western blot (Fig. 3e, f).

### Overexpression of ALDH3A1 inhibits the migration and invasion of OSCC cells under chronic stress

**Fig. 4:**
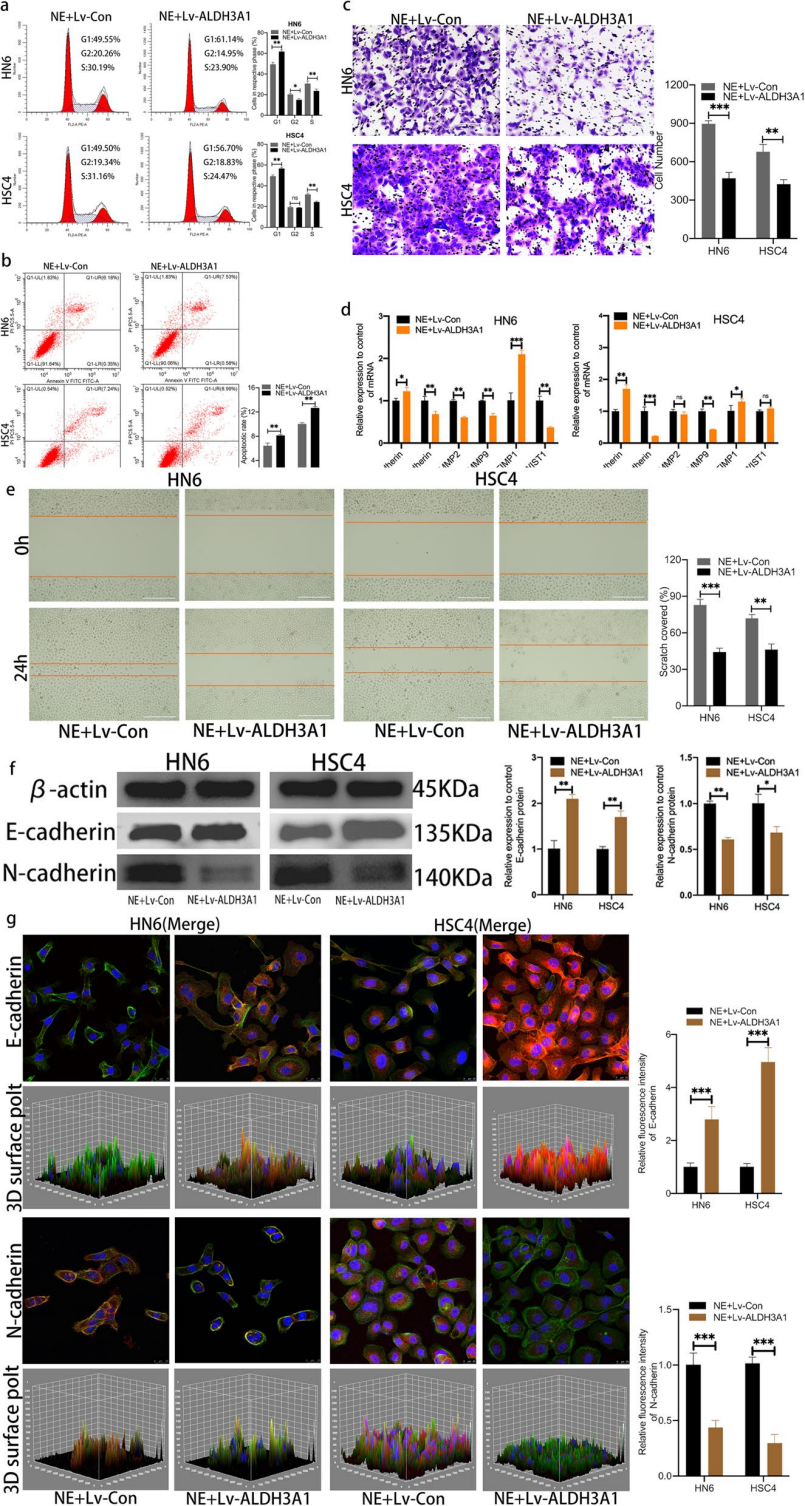
Overexpression of ALDH3A1 inhibited the migration and invasion of OSCC cells under chronic stress. **a, b** Rate of cell cycle and apoptosis in both groups was determined by flow cytometry. **c** Cell migration ability of HN6 and HSC4 cell lines in NE + Lv-con and NE + Lv-ALDH3A1 groups was determined using wound-healing assays. Scale bars, 400 μm. **d** mRNA expression of tumor regulatory factors and EMT-related markers in OSCC cells in both groups. **e** Cell invasion ability of HN6 and HSC4 cells was determined using trans-well assays. Scale bars, 200 μm. **f** Protein levels of E-cadherin and N-cadherin in both groups of OSCC cells (The blots were cropped and the full-length raw blots are provided in the supplementary). **g** Confocal laser scanning microscope images of immunofluorescent staining of E-cadherin and N-cadherin in OSCC cells, blue for nuclear, green for cytoskeleton and red for protein, Scale bars, 25 μm. Data are expressed as mean ± SD (*n* = 3); *, *P* < 0.05; **,* P* < 0.01; ***, *P* < 0.001

To clarify the function of ALDH3A1 in OSCC cells under chronic stress, the Lv-Con and Lv-ALDH3A1 OSCC cells were treated with 10um NE. The flow cytometry analysis showed that the percentage in S-phase were evidently decreased and the percentage of apoptotic were significantly increased in NE + Lv-ALDH3A1 group compared with the NE + Lv-Con group (Fig. 4a, b). Transwell assays and wound healing test showed that Lv-ALDH3A1 suppressed the invasive ability and inhibited the migration of OSCC cells under chronic stress (Fig. 4c, e). In addition, we detected the expression of tumor managing factors and EMT markers. The results of RT-PCR demonstrated that N-cadherin, MMP2, MMP9 and TWIST1 were down-regulated, while E-cadherin and TIMP1 were up-regulated in NE + Lv-ALDH3A1 group (Fig. 4d). Western blot results indicated that highly-expressed ALDH3A1 decreased expression of N-cadherin but increased expression of E-cadherin in OSCC cells under chronic stress (Fig. 4f). Furthermore, immunofluorescence staining displayed a similar result with western blot (Fig. 4g, sFig 2). Together, these data shed light on the potential role of ALDH3A1 in inhibiting OSCC cell proliferation, migration and invasion.

### Under CRS, overexpression of ALDH3A1 restrains tumor growth in xenograft models of OSCC

**Fig. 5:**
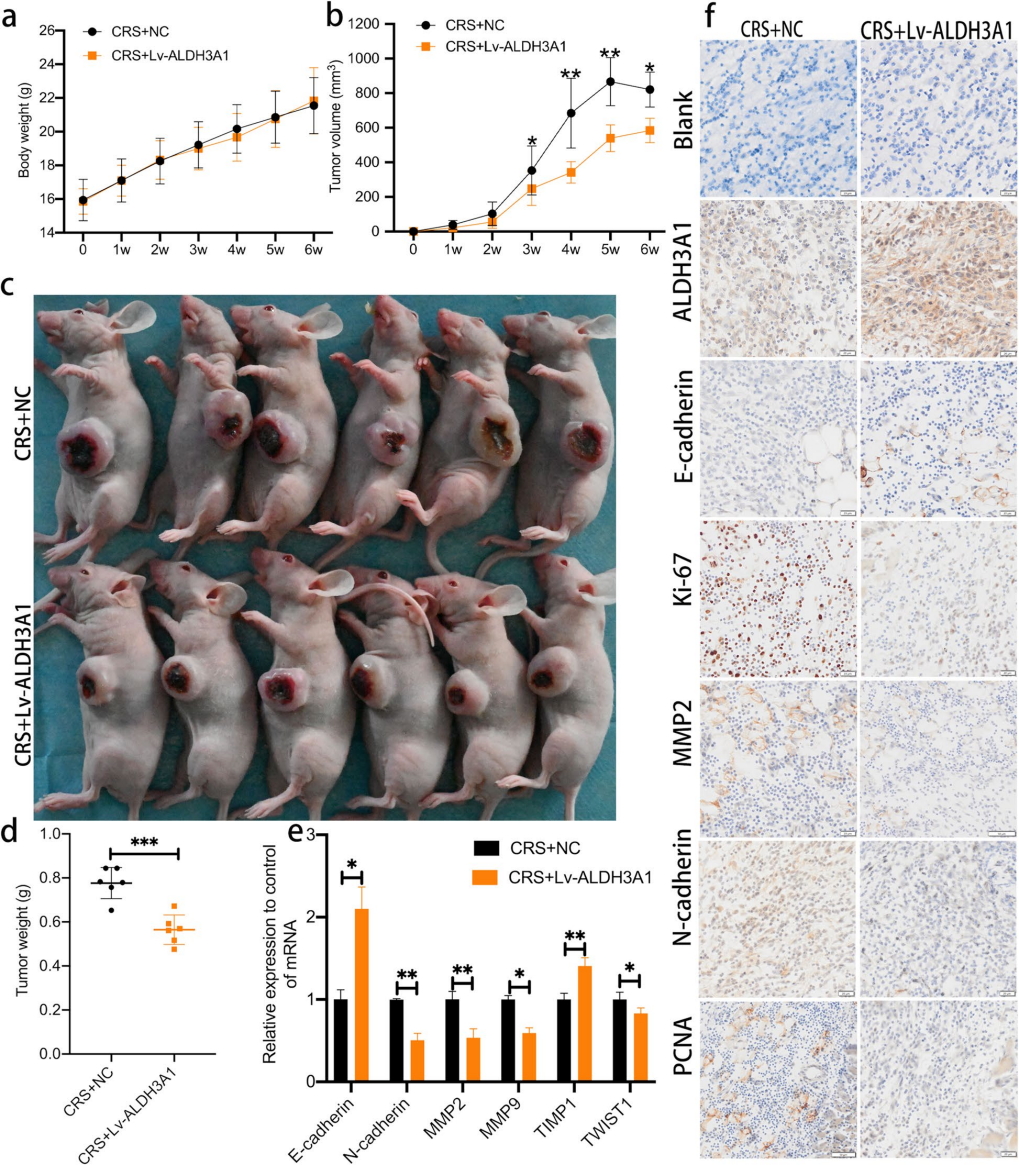
With CRS, overexpression of ALDH3A1 restrain tumor growth in Xenograft models of OSCC. **a** Body weight curve of CRS + NC and CRS + Lv-ALDH3A1 groups. **b** Growth curves of tumor volume**. c** Tumor nodules of mice xenografts. **d** Tumor tissues weight were determined. **e** mRNA expression of tumor regulatory factors and EMT-related markers in tumor tissues were determined by RT- PCR. **f** Representative images of ALDH3A1, proliferation markers, tumor regulatory factors and EMT-related markers protein levels in tumors were assessed by immunohistochemical staining. Scale bar, 20 μm. Data are expressed as mean ± SD; *, *P* < 0.05; **,* P* < 0.01; ***, *P* < 0.001

We founded the subcutaneous graft tumor model to evaluate the role of ALDH3A1 on OSCC growth in vivo. Nude mice were randomly parted into two different groups. All mice began chronic restraint stress one day before the injection of OSCC cells and stopped after 4 weeks. Experimental group were injected subcutaneously with LV-ALDH3A1 cells, while the NC group of mice were injected with negative control cells. Both groups were of similar weight before and after treatment (Fig. 5a). However, 3 weeks after HN6 cell injection, the tumor of CRS + LV-ALDH3A1 group was meaningfully smaller than that of CRS + Group (Fig. 5b). At 6 weeks, tumor tissues were excised for further analysis. The results showed that the tumor weight of mice in CRS + LV-ALDH3A1 group was lower than that of mice in CRS + NC Group (Fig. 5c, d). Next, we performed RT- PCR and immunohistochemical (IHC) staining with tumor tissues. RT-PCR results showed that the mRNA levels of N-cadherin, MMP2, MMP9 and TWIST1 were up-regulated in CRS + LV-ALDH3A1 group, while the mRNA levels of E-cadherin and TIMP1 were down-regulated in CRS + LV-ALDH3A1 group (Fig. 5e). IHC displayed that the expression of Ki-67, MMP2, N-cadherin and PCNA were meaningfully decreased in CRS + LV-ALDH3A1 group, while the expression of ALDH3A1 and E-cadherin in CRS + LV-ALDH3A1 group was higher than that in CRS + NC Group (Fig. 5f). Thus, these findings demonstrated that overexpression of ALDH3A1 can inhibit tumor growth in xenograft models of OSCC under chronic restraint stress.

### CRS influences mitochondrial metabolism of OSCC by modulating ALDH3A1

**Fig. 6:**
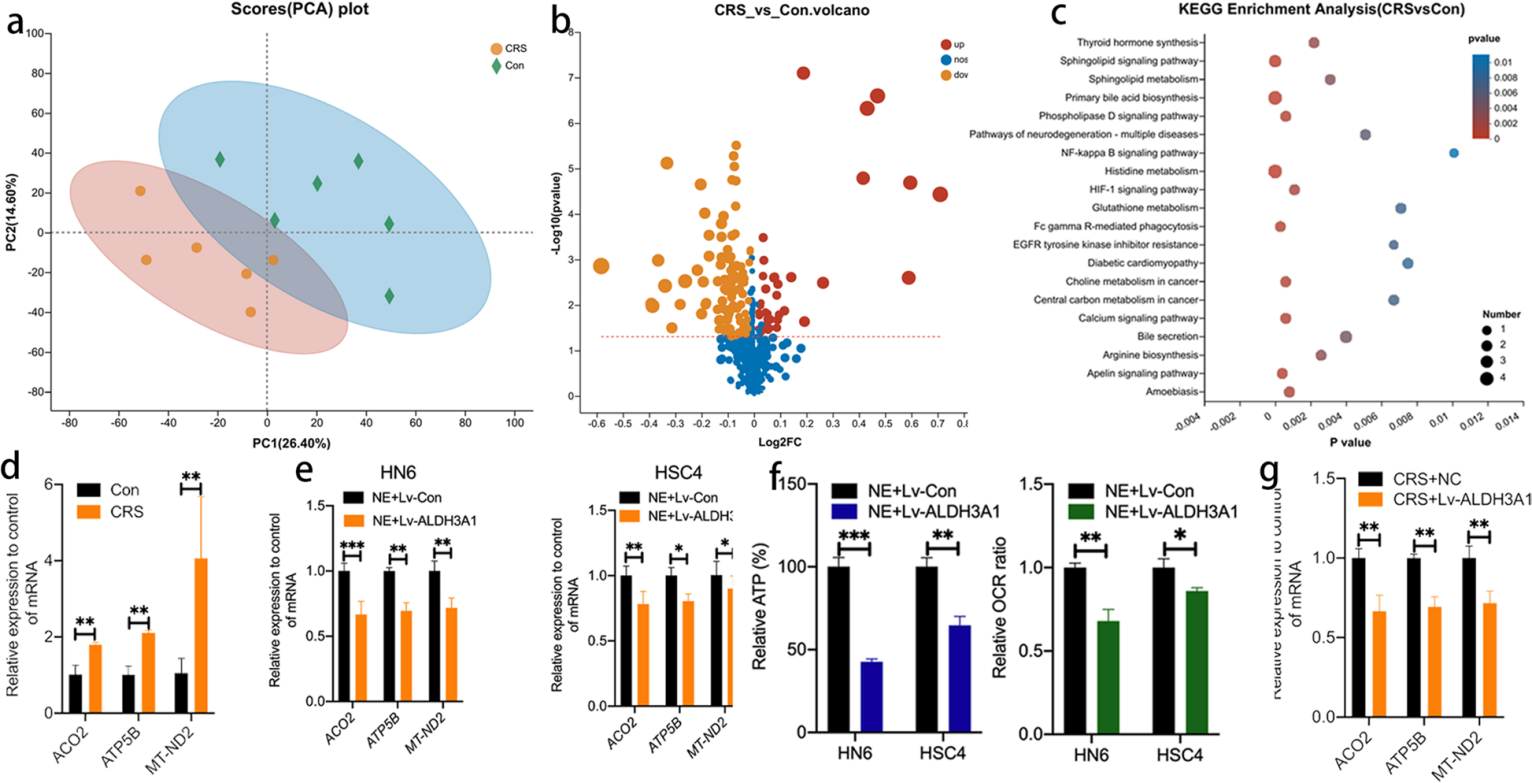
CRS influence mitochondrial metabolism of OSCC by modulating ALDH3A1. **a** PCA score plot of tumor tissue-based metabolite profiling between the Con and CRS groups. **b** volcano plot of differentially metabolites (DMs). The threshold set for DMs was fold-changes (FC) > 2 and *p* < 0.05, 92 metabolites are downregulated (yellow) and 28 metabolites are upregulated (red). **c** KEGG pathway enrichment analysis of DMs. **d** mRNA expression of key mitochondrial metabolism factors in mice xenografts with Con and CRS groups. **e** mRNA expression of key mitochondrial metabolism factors in OSCC cells. **f** Relative levels of ATP production and OCR in HN6 and HSC4. **g** mRNA expression of key mitochondrial metabolism factors in mice xenografts with CRS + NC and CRS + Lv-ALDH3A1 groups. Data are expressed as mean ± SD; *, *P* < 0.05; **,* P* < 0.01; ***, *P* < 0.001

To track metabolic changes in OSCC cells under the CRS state, we used LC–MS /MS for untargeted metabolic analysis of tumor tissues. Principal component analysis (PCA) showed significant differences between CRS group and Con group (Fig. 6a). The 130 different metabolites were all identified, including 92 metabolites were decreased and 28 metabolites were increased in CRS group (Fig. 6b). The KEGG pathway enrichment analysis [24] revealed that 20 metabolite-related pathways, including mitochondrial metabolism, were significantly varied between the CRS and Con Group (Fig. 6c). We further detected the expression of genes related to mitochondrial metabolism in tumor tissues by RT-PCR [25]. The result showed that ACO2, ATP5B, and MT-ND2 mRNA levels were observably increased in CRS group when compared with Con group (Fig. 6d). These data have suggested that CRS induces changing of mitochondrial metabolism in OSCC. To explore whether ALDH3A1 plays a regulatory role of mitochondrial metabolic in OSCC cells under chronic stress, we performed RT-PCR, ATP analysis, and oxygen consumption rate (OCR) analysis in NE + LV-ALDH3A1 and NE + Lv-Con OSCC cells. The results of RT-PCR demonstrated that ACO2, ATP5B, and MT-ND2 were down-regulated in NE + Lv-ALDH3A1 group compared with those in NE + Lv-Con group (Fig. 6e). ATP and OCR analysis showed that ATP consumption and OCR of OSCC cells were significantly reduced in NE + LV-ALDH3A1 group (Fig. 6f, sFig 3). Moreover, the RT-PCR of tumor tissues showed that mRNA level of ACO2, ATP5B, and MT-ND2 were lower in CRS + Lv-ALDH3A1 than those in CRS + NC group (Fig. 6g).

## Discussion

In this article, we identified the potential pathway and molecular mechanism of CRS regulating the progress of OSCC. Here, we have revealed that regulating ALDH3A1 is the key pathway for CRS to promote OSCC. Additionally, we demonstrated that ALDH3A1 plays a crucial role in reprogramming mitochondrial metabolism and overexpression of ALDH3A1 inhibited the growth and ATP accumulation of OSCC cells.

Since a groundbreaking study focused on the importance of psychology and behavior in the development of the disease [26], many subsequent studies have focused on the influence of chronic stress on tumor progression [27, 28]. Previous studies have shown a positive association between chronic stress and carcinoma progression [13, 29]. In addition, researches have shown that chronic stress can facilitate the increase of catecholamines by activating HPA [30]. Our study found that CRS promotes OSCC growth, invasion, and metastasis, and significantly increases NE level.

Recently, the effect of nervous system adjustment in the occurrence and progression of cancer has been widely researched and received [31–33]. Sympathetic and parasympathetic excitation stimulates the release of neurotransmitters such as acetylcholine, epinephrine, and substance P. These neurotransmitter stimuli cause downstream signal transduction by activating specific receptors on the cell surface. There was new proof that chronic stress hormone is a risk factor for cancer development and is considered as a marker of malignant progression of tumors [34, 35]. High levels of stress hormones contributed to carcinogenesis by inducing accumulation of DNA damage, increasing p53 degradation, or other related pathways [30]. Previous study reported that NE induces the advancement and diversion of gastric cancer via ADRB2 signaling pathway [7]. Consistent with previous studies [36, 37], we found that HN6 and HSC4 cultured with NE showed increased proliferation and migration ability compared with the Con group. Distant metastasis is a typical malignant behavior of tumors, and we found that stress response hormone can promote the EMT of OSCC cells. In conclusion, this study revealed that CRS facilitates the growth, motion and invasion of OSCC cells through stress response hormones.

We then further found that CRS and stress response hormone downregulated the expression of ALDH3A1. ALDH3A1 has various biological effects, including maintenance of hematopoietic stem cells, regulation of cell radiation, proliferation and so on [38, 39]. Recent years, a large number of studies have found that ALDH3A1 can be considered as a marker for predicting tumor prognosis and associated with poor clinical outcomes of various tumors [40, 41]. In oral mucosa, ALDH3A1 was detected to promote the anti-oxidation and anti-injury ability of epithelial cells, inhibit inflammatory reaction and maintain DNA integrity [15, 42]. Thus, we explored the effect of ALDH3A1 in OSCC development. In this section, we acquired that overexpression of ALDH3A1 can reverse the effects of stress response hormone on migration and invasion of OSCC cells, this data in accordance with those reported in this research [19], which demonstrated that overexpression of ALDH3A1 in OSCC cells significantly inhibits cell proliferation and invasion. However, Wu et al. [18] demonstrated that highly-expressed ALDH3A1 is correlated with gastric cancer malignant progression. Oral squamous cell carcinoma originates from genetic mutation in the upper layer of oral mucosa, and the high expression of ALDH3A1 in normal oral mucosal epithelium may be responsible for these adverse outcomes. Meaningfully, our results suggest that under chronic stress overexpression of ALDH3A1 can restrain the tumorigenic potential of OSCC cells.

The ALDH3A subfamily contains ALDH3A1 and ALDH3A2 enzymes, which are relevant to the oxidation of fats and aromatic aldehydes, as well as the generation of NADPH. Study has shown that ALDH3A1 may be involved in regulating REDOX dependent signal transduction pathways during tumor progression [43]. Terzuoli et al. reported that ALDH3A1 can affect the stemness and EMT of melanoma and lung tumors by regulating the metabolism of tumor cells [23]. Mitochondrial metabolism plays a primary role in conditioning whether immune response promotes or suppresses cancer [44]. Another study found that TLR4 activation reprograms mitochondrial metabolism to meet the promptly increasing energy needs of cells in an inflammatory condition [45]. Tlr4 induced inflammatory cytokine production and mitochondrial reprogramming require the involvement of STAT3 [46]. ALDH3A1 inhibits TLR4 activation, leading to the phosphorylation of STAT3, which alters mitochondrial metabolism [15]. Our study found that CRS induces metabolic changes in OSCC through mitochondrial reprogramming. Furthermore, mitochondrial metabolism was inhibited in OSCC cells by overexpressing ALDH3A1 under chronic stress. These outcomes suggest that regulation of mitochondrial metabolism is one of the underlying mechanisms by which ALDH3A1 inhibits the carcinogenic potential of OSCC cells.

## Conclusion

In summary, we demonstrate that inhibition of ALDH3A1 expression by stress response hormones is a key pathway by which CRS enhances the oncogenic potential of OSCC cells. Furthermore, the regulating of mitochondrial metabolism may be involved in this process. Our study affords a new insight into the molecular mechanism by which CRS promotes oral squamous cell carcinoma development. However, the limitation of our study is the establishment of xenograft models in nude mice, so the immune microenvironment is not involved. Therefore, our next study may focus on the role of CRS in OSCC immunomodulation.

### Supplementary Information

**Additional file 1:** **Supplementary Materials and Methods. Supplementary Table 1.** Primer sequences for qPCR amplification of specific genes. **Supplementary Figure 1.** Immunofluorescent staining of EMT-related markers in OSCC cells with Con and NE groups. **Supplementary Figure 2.** Immunofluorescent staining of EMT-related markers in OSCC cells with NE+Lv-Con and NE+Lv-ALDH3A1 groups.** Supplementary Figure 3.** Relative levels of ATP production and OCR of Lv-Con and Lv-ALDH3A1 HN6 and HSC4 without NE treatment. **Supplementary of figure 2g.** The original pictures of western blots of figure 2g. **Supplementary of figure 3d.** The original pictures of western blots of Figure 3d. **Supplementary of figure 3f.** The original pictures of western blots of Figure 3f. **Supplementary of figure 4f.** The original pictures of western blots of Figure 4f.

## Abbreviations

CRS: Chronic restraint stress
OSCC: Oral squamous cell carcinoma
NE: Norepinephrine
ALDH3A1: Acetaldehyde dehydrogenase 3A1
EMT: Epithelial mesenchymal transition
HPA: Hypothalamic–pituitary–adrenal
