# Supplementary material for: Chronic restraint stress promotes oral squamous cell carcinoma development by inhibiting ALDH3A1 via stress response hormone
Source: BMC Oral Health. 2024 Jan 8;24:43. doi: 10.1186/s12903-023-03787-1 (PMC10773021; doi:10.1186/s12903-023-03787-1)
Supplement: Supplementary file 1 — Additional file 1: Supplementary Materials and Methods. Supplementary Table 1. Primer sequences for qPCR amplification of specific genes. Supplementary Figure 1. Immunofluorescent staining of EMT-related markers in OSCC cells with Con and NE groups. Supplementary Figure 2. Immunofluorescent staining of EMT-related markers in OSCC cells with NE+Lv-Con and NE+Lv-ALDH3A1 groups. Supplementary Figure 3. Relative levels of ATP production and OCR of Lv-Con and Lv-ALDH3A1 HN6 and HSC4 without NE treatment. Supplementary of figure 2g. The original pictures of western blots of figure 2g. Supplementary of figure 3d. The original pictures of western blots of Figure 3d. Supplementary of figure 3f. The original pictures of western blots of Figure 3f. Supplementary of figure 4f. The original pictures of western blots of Figure 4f. [file 12903_2023_3787_MOESM1_ESM.docx]

**Supplementary Materials and Methods**

**Liquid chromatography–mass spectrometry (****LC–MS)**

Briefly, each sample was mixed with pre-chilled methanol/acetonitrile/water (2:2:1, v/v/v) mixture and then vortexed for 30 s, and stored at -20℃ for 20 min, and then centrifuged for 20 min at 14000 g under 4℃. The supernatant was collected and dried in vacuum and stored at −80℃. Then, the dried metabolites were re-dissolved with acetonitrile/water (1:1) solvent, and then centrifuged for 15 min at 14,000 g and 4℃, and the supernatant was collected and analyzed.

Samples after pretreatment were separated using an ultra-performance liquid chromatography (UPLC) system (Agilent Technologies Inc. CA, UAS) and a 1.7 μm HILIC column (2.1mm × 100 mm, Waters, Milford, MA). After separation, the samples for positive ion mode and negative ion mode that connected to UPLC system via Electrospray ionization (ESI) port were analyzed using Triple TOF™ 5600 plus Mass Spectrometer (Applied Biosystems, CA, USA).

**Quantitative real‑time polymerase chain reaction (****RT‑PCR)**

RT-PCR was performed to measure levels in the mRNA expression of the following genes: ADRB2, ALDH3A1, E-cadherin, N-cadherin, MMP2, MMP9, TIMP1, Twist1, ACO2, ATP5B and MT-ND2. The total RNA extraction kit (Beyotime, China) was used for total RNA extraction from the tissue samples and treated cells. cDNA was synthesized from total RNA using a PrimeScript RT reagent kit with gDNA Eraser (MedChemExpress, China). The ABI 7300 system (Applied Biosystems, USA) as used to perform Real-time PCR by the SYBR Premix ExTaq kit (MedChemExpress, China). We used β-actin as the reference and the 2^−△△CT^ method to determine the relative expression of the target genes. Primer sequences are presented in supplementary Table1.

**Western blot assay**

The total protein was extracted by using total Protein Extraction Kit (Beyotime, China). The proteins were separated by 10% or 8% SDS-PAGE (Beyotime, China) and transferred onto polyvinylidene difluoride (PVDF) membranes (Millipore, Bedford, MA). The membranes were incubated with the following primary antibodies: anti-β-Actin (1:1000; 4970s, Cell Signaling); anti-ALDH3A1 (1:1000; ab227694, abcom); anti- N-cadherin (1:1000; 13116T, Cell Signaling) and anti- E-cadherin (1:1000; 3195T, Cell Signaling). Then the membranes were incubated with secondary horseradish peroxidase-conjugated goat anti-rabbit antibody (1:3000; Cell Signaling) and visualized using a Bio-Rad Imaging System (Bio-Rad, Hercules, CA, USA). The protein bands were analyzed using ImageJ. The expression of β-Actin was used as internal control. Some blots, especially β-actin blots, were cut prior to hybridisation with antibodies and highly expressed, which makes the image appear to be without portion marker. The full-length western blot compliance with the digital image and integrity policies, all images were unprocessed files.

**Cell proliferation assay**

Briefly, Cells (1 × 10^3^ per well) were seeded into 96-well plates. After treatment, the absorbance at 450 nm was detected at different-time points according to the instructions. The absorbance values were measured using a microplate reader (SpectraMAX iD5, USA).

**Cell apoptosis assays**

The Annexin V-FITC/PI Apoptosis Detection Kit used to evaluate the apoptosis rate according to the instructions. Cells (4 × 10^5^ per well) were seeded into 6-well plates. Following treatment, the cells were collected, washed with PBS. Then, the cells were resuspended in 500μL PBS. Next, Annexin V-FITC and PI were added to the cells and incubated at 37°C for 15min. Cells were analyzed by flow cytometry (BD FACSCanto).

**Cell cycle analysis**

Cells (4 × 10^5^ per well) were seeded into 6-well plates. Following treatment, the cells were collected, washed with PBS, and resuspended in 100μL PBS. Then, the cells were added slowly 500μl 75% precooled ethanol. Next, RNase A solution was added to the cells were incubated at 37°C for 30min, and PI was added and incubated for 30min at 37°C. The DNA content was detected by flow cytometry (BD FACSCanto). The data (percentage of cells in the G1 phase, the S phase, and the G2 phase) was analyzed by Cell Quest software (Becton Dickinson, Franklin Lakes, NJ).

**Cell migration and invasion assays**

Migration of cells was examined using scratch wound healing assays. Briefly, cells were seeded in 6-well plates and cultured to confluence. Images of wounds were captured at 0, and 24 h after scratching with a sterile 200 µl yellow pipette tip. Migration distance was measured at three different positions. Invasion ability of cells was examined using transwell assays. Cells in serum free culture medium were loaded in the upper chamber that was precoated with Matrigel (BD Biosciences). Complete medium containing serum was added to the bottom wells of the transwell chambers (BD Biosciences). After incubation for 24 hours, cells were fixed and stained with Trypan Blue (Beyotime, China). Cells that had migrated to the lower membrane surface were counted.

**Immunofluorescence**

Cells on sterile cover slips were fixed with 4% paraformaldehyde for 20 min, permeabilized using 0.5% Triton X-100 for 10 min, and then blocked with 5% sheep serum for 1 hour. The cells were incubated with anti-ALDH3A1 (1:200; ab227694, abcom), anti- N-cadherin (1:200; 13116T, Cell Signaling) and anti- E-cadherin (1:200; 3195T, Cell Signaling) primary antibody for overnight at 4°C, and then with a goat anti-rabbit DyLight 594 secondary antibody (1:500; Beyotime, China) for 1 hour at 37°C. Cytoskeleton and nuclear staining was performed with phalloidin and 4′,6-diamidino-2-phenylindole (DAPI), respectively. The cells were analyzed using a confocal laser scanning microscopy (Nikon, Tokyo, Japan).

**Immunohistochemistry**

Briefly, the sections were dewaxed and rehydrated with xylene and graded ethanol, respectively. Then, the endogenous horseradish peroxidase activity was blocked using 3% hydrogen peroxide. The sections were blocked after antigen retrieval. Subsequently, the sections were incubated overnight at 4 °C with primary antibodies and then with specific secondary antibodies.

**Supplementary Table 1**

Primer sequences for qPCR amplification of specific genes:

| Genes |  | Sequence (5’→3’) |
| --- | --- | --- |
| β-Actin | forward | AGAAAATCTGGCACCACACCT |
|  | reverse | GATAGCACAGCCTGGATAGCA |
| ADRB2 | forward | TTGCTGGCACCCAATAGAAGC |
|  | reverse | CAGACGCTCGAACTTGGCA |
| ALDH3A1 | forward | TGGAACGCCTACTATGA GGAG |
|  | reverse | GGGCTTGAGGACCACT GAG |
| E-cadherin | forward | TGGACCGAGAGAGTTTCCCT |
|  | reverse | CAAAATCCAAGCCCGTGGTG |
| N-cadherin | forward | GGGAAATGGAAACTTGATGGC |
|  | reverse | AGCTTCTCACGGCATACACC |
| MMP2 | forward | TGGACTTAGACCGCTTGGCT |
|  | reverse | TCCTTGGGGCAGCCATAGAA |
| MMP9 | forward | TCTATGGTCCTCGCCCTGAA |
|  | reverse | CATCGTCCACCGGACTCAAA |
| TIMP1 | forward | GTGGCACTCATTGCTTGTGG |
|  | reverse | GTGGCACTCATTGCTTGTGG |
| Twist1 | forward | GAACACTCGTTTGTGTCCCC |
|  | reverse | CAGTGGCTGATTGGCAAGAC |
| ACO2 | forward | AGCCCAACGAGTACATCCAT |
|  | reverse | TCTTCTCCGAGAGTGTCAGC |
| ATP5B | forward | TGTTTGCTGGTGTTGGTGAG |
|  | reverse | GAGCACCAGGTGGTTCATTC |
| MT-ND2 | forward | CCCAACCCGTCATCTACTCT |
|  | reverse | AAATCAGTGCGAGCTTAGCG |

**Supplementary Figure 1**

**
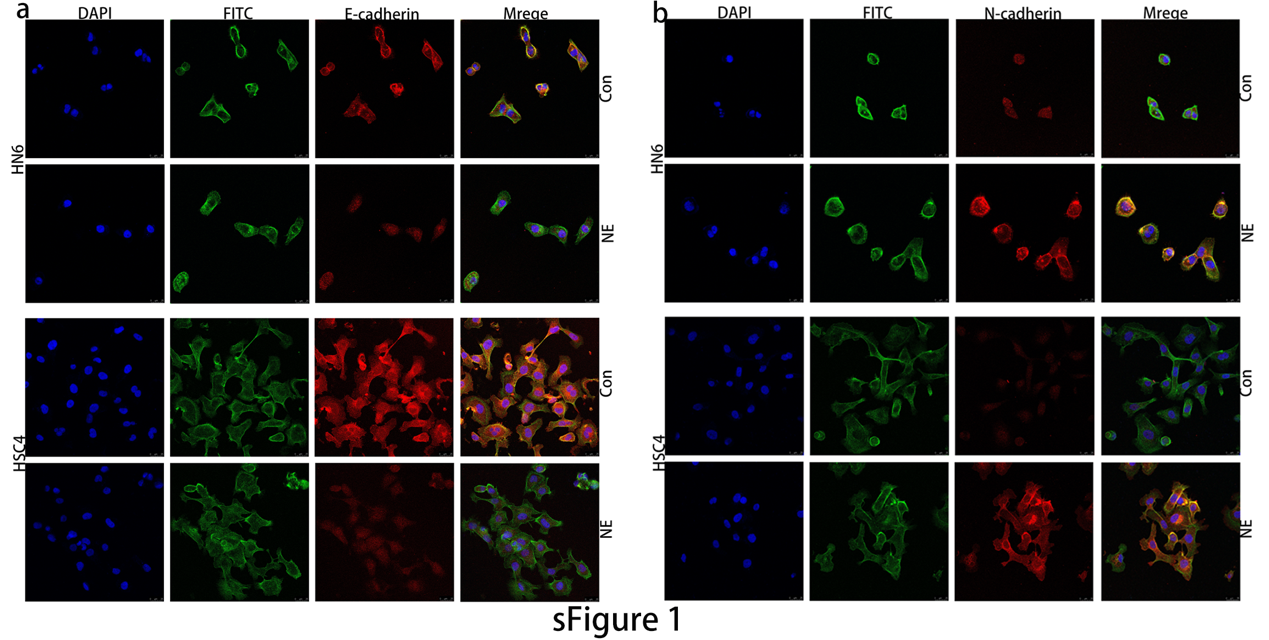
**

**Supplementary Figure 1** Immunofluorescent staining of EMT-related markers in OSCC cells with Con and NE groups. **a** Confocal laser scanning microscope images of immunofluorescent staining of E-cadherin, blue for nuclear, green for cytoskeleton and red for protein. **b** Confocal laser scanning microscope images of immunofluorescent staining of N-cadherin, blue for nuclear, green for cytoskeleton and red for protein. Scale bars, 25μm.

**Supplementary Figure 2**


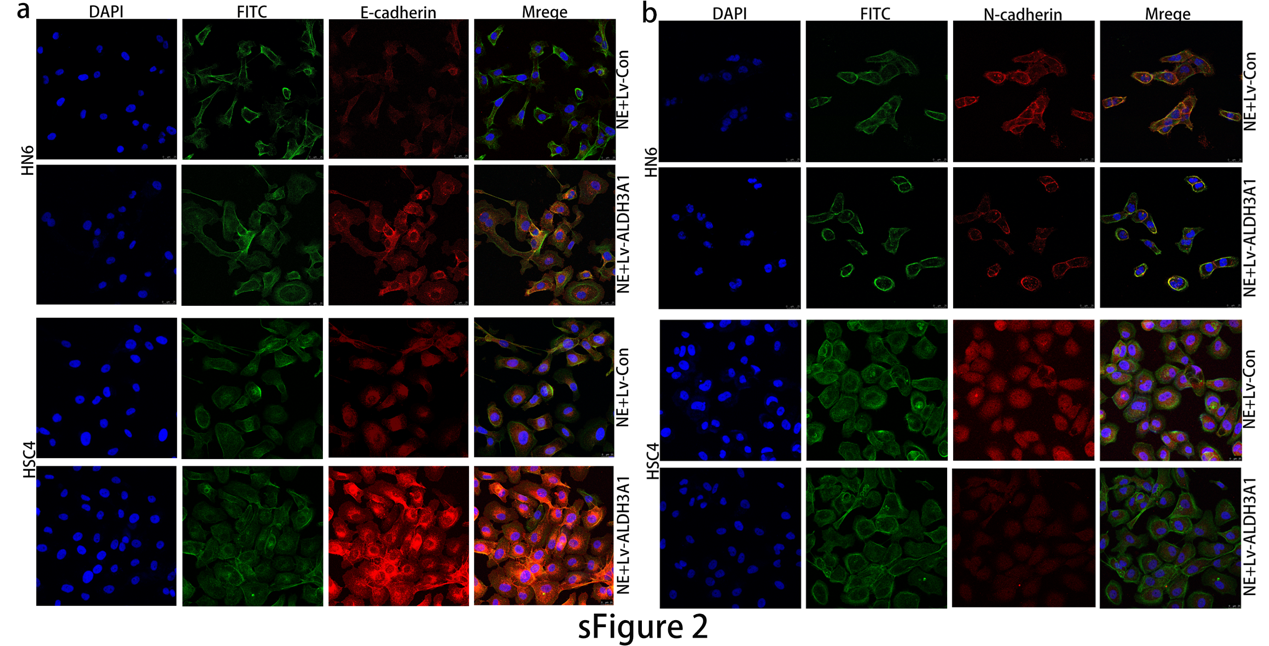


**Supplementary Figure 2** Immunofluorescent staining of EMT-related markers in OSCC cells with NE+Lv-Con and NE+Lv-ALDH3A1 groups. **a** Confocal laser scanning microscope images of immunofluorescent staining of E-cadherin, blue for nuclear, green for cytoskeleton and red for protein. **b** Confocal laser scanning microscope images of immunofluorescent staining of N-cadherin, blue for nuclear, green for cytoskeleton and red for protein. Scale bars, 25μm.

**Supplementary Figure 3**


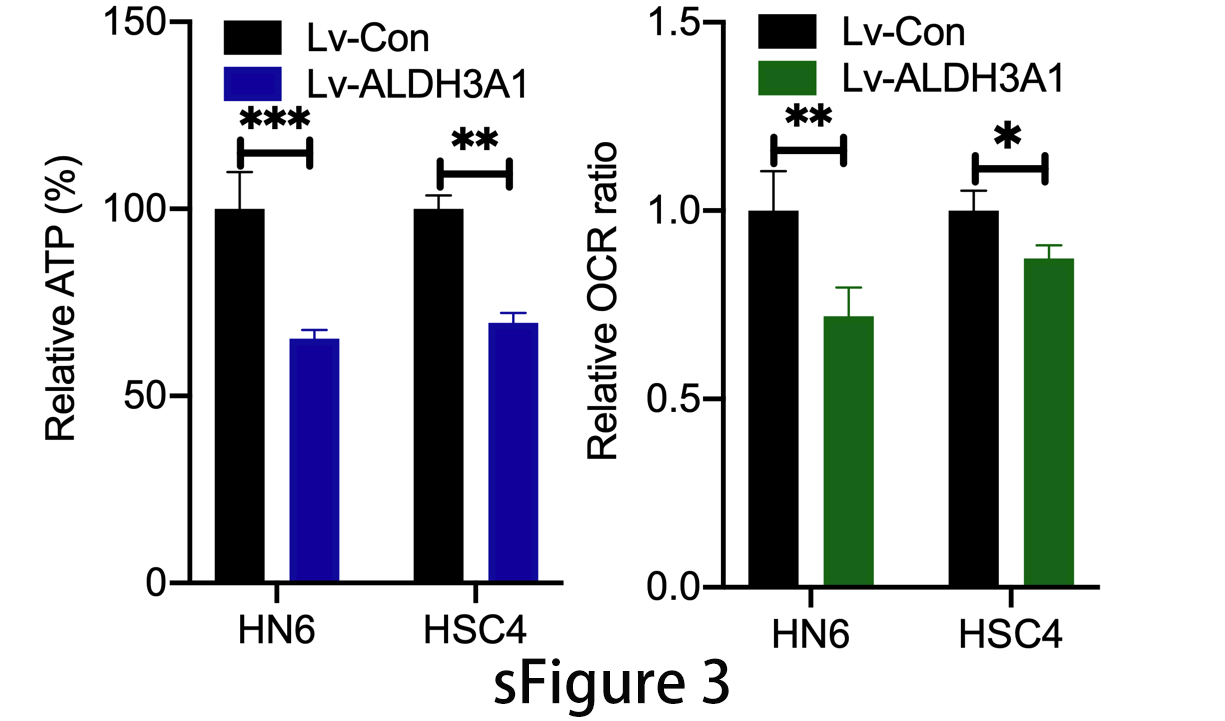


**Supplementary Figure 3** Relative levels of ATP production and OCR of Lv-Con and Lv-ALDH3A1 HN6 and HSC4 without NE treatment

**Original** **pictures of western blots in this study:**

**Supplementary of figure 2g**


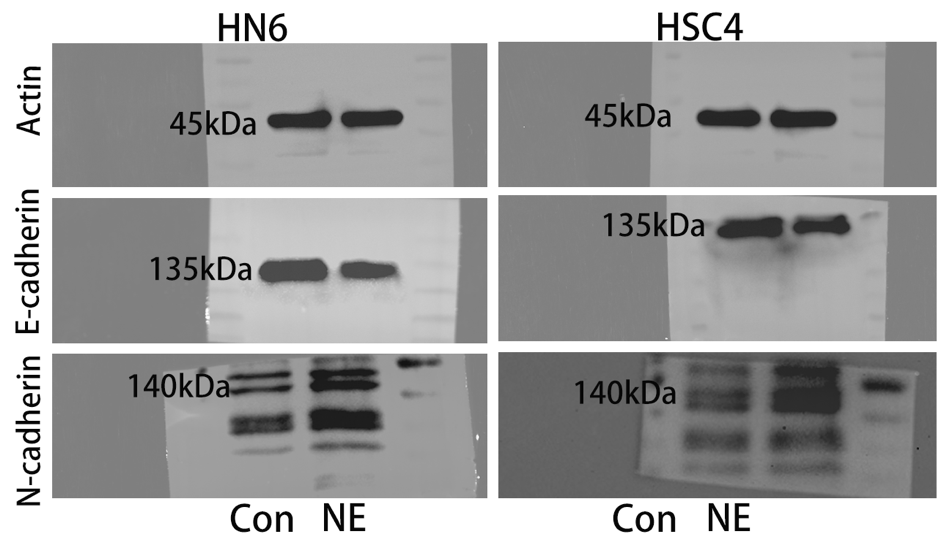


**Supplementary of figure 2g:** The original pictures of western blots of figure 2g

**Supplementary of figure 3d**


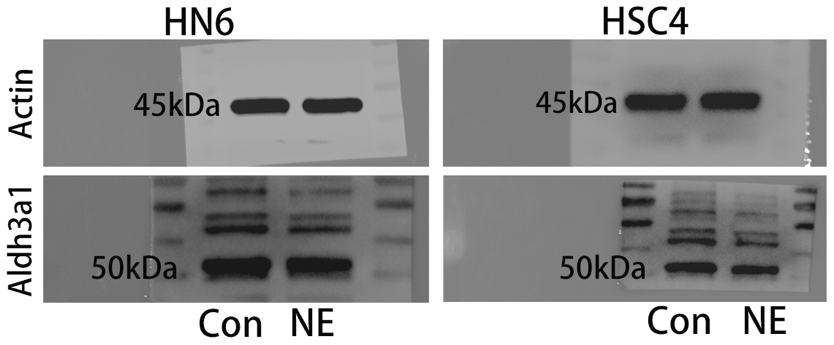


**Supplementary of figure 3d:** The original pictures of western blots of Figure 3d

**Supplementary of figure 3f**


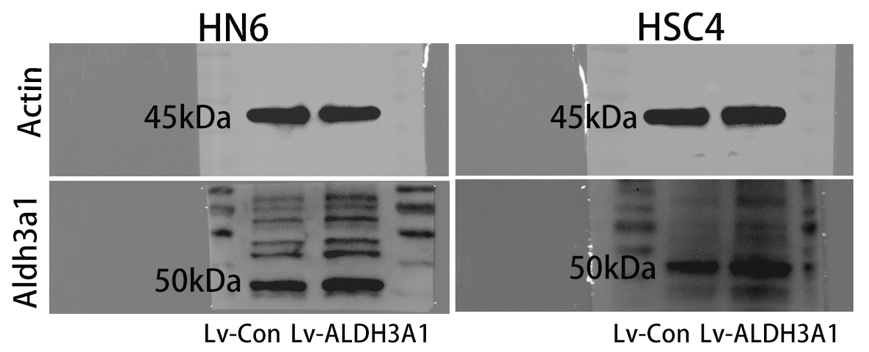


**Supplementary of figure 3f:** The original pictures of western blots of Figure 3f

**Supplementary of figure 4f**

**
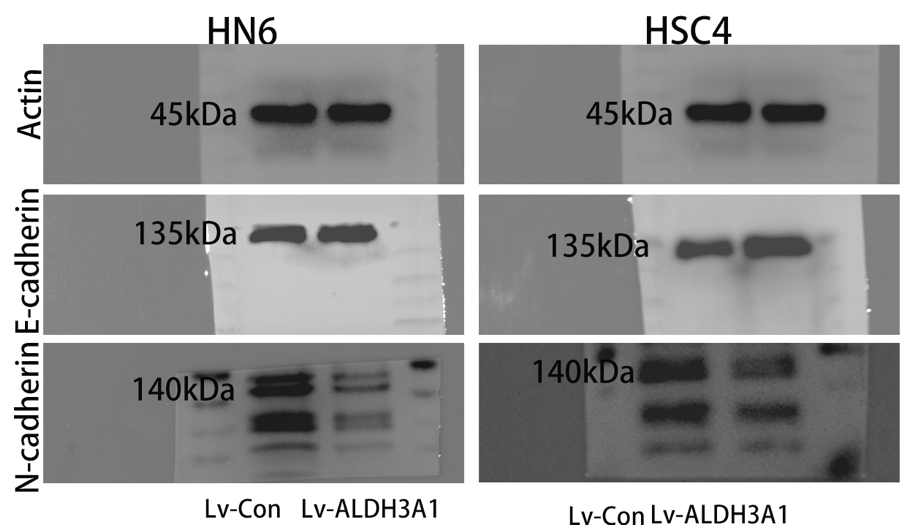
**

**Supplementary of figure 4f:** The original pictures of western blots of Figure 4f
